# Contribution of dermal-derived mesenchymal cells during liver repair in two different experimental models

**DOI:** 10.1038/srep25314

**Published:** 2016-04-29

**Authors:** Li Tan, Tingyu Dai, Dengqun Liu, Zelin Chen, Liao Wu, Li Gao, Yu Wang, Chunmeng Shi

**Affiliations:** 1 Institute of Combined Injury, State Key Laboratory of Trauma, Burns and Combined Injury, Chongqing Engineering Research Center for Nanomedicine, College of Preventive Medicine, Third Military Medical University, 30 Gaotanyan Road, Chongqing 400038, China; 2 Department of Hematology, Second Affiliated Hospital, Third Military Medical University, Xinqiao Road, Chongqing, 400037, China

## Abstract

Progressive liver disease is a major health issue for which no effective treatment is available, leading to cirrhosis and orthotopic liver transplantation. However, the lack of availability of donor organs and other adverse factors including rejection limit its extensive clinical application. Cell-based therapy using mesenchymal stem/stromal cells (MSCs) may represent an attractive therapeutic option. Dermal-derived mesenchymal cells (DMCs) are attractive as one of the abundant sources from which to isolate mesenchymal cells for therapeutic applications and can be easily accessed with minimal harm to the donor. In this study, we used two different animal models to investigate potential therapeutic effect of DMCs transplantation in liver injury. We found that DMCs administration alleviated liver fibrosis and restored the liver function in fibrotic mice induced by CCl_4_. Furthermore, in an acute irradiation induced damage model, a unique population of DMCs could engraft into the liver tissue for a long period, exhibiting the phenotype of both mesenchymal cells and macrophage cells, and improve the survival of mice exposed to 8 Gy lethally total-body irradiation. These discoveries provide important evidence that DMCs therapy has a beneficial effect on liver injury, and provide new insight into liver injury therapy depending on the alternative cells.

The liver is a critically important organ that possesses an immense capacity to regenerate and mounts a biological defense against foreign injury. However, when the injury progresses into a state of functional impairment, it can overwhelm or even inhibit the intrinsic regenerative potential, leading to severe consequences that include liver failure or death^1^. Currently, the outcomes of end-stage liver disease display a significant mortality rate and liver transplantation is the primary treatment for it with a 4-year survival rate of 70% or greater for most clinical indications^2^,^3^. However, the lack of availability of donor organs and other adverse factors including rejection limit its extensive clinical application^4^.

Recently, mesenchymal stem/stromal cell (MSC)-based therapy has been extensively investigated in the area of regenerative medicine for different organs because of its ability to differentiate and transdifferentiate into various tissue types, stimulate regeneration, and repair damaged tissue/organs. Researchers have shown that bone marrow–derived mesenchymal stem cells effectively rescued experimental liver failure and contributed to liver regeneration^5^, and this offers a potentially alternative therapy to organ transplantation for treatment of liver diseases. Bone-marrow-derived MSCs improve liver function in patients with liver cirrhosis as evidenced by phase I clinical trials^6^,^7^,^8^,^9^. Compared to MSCs, different site-specific mesenchymal cells also show regenerative and immunomodulatory properties for a cell therapy candidate. Skin, which consists of epidermis, dermis and appendages, is known as the largest organ in mammals including human. Previous studies have proved that the presence of abundant MSC-like cell populations existed in the dermis^10^,^11^. Due to their easy accessibility, these dermal derived cells were considered to be the source of autologous cell therapy for various diseases. Our previous studies have shown that the transplanted dermal derived mesenchymal cells (DMCs) promoted survival and wound healing in rats with combined radiation and wound injury^12^. We further proved that DMCs therapy decreased the incidence and severity of acute GVHD and improved the survival of sepsis in mice^13^,^14^. Several studies have reported that fibroblast form skin dermis can differentiate into cells that displayed hepatic characteristics and function^15^,^16^. However, it remains to be clarified if dermal derived mesenchymal cells are able to directly contribute beneficial effect to liver injury. Furthermore, their ability to respond to different type of liver injury has not been investigated.

Thus, in the current study, we used two different animal models to investigate potential therapeutic effect of DMCs transplantation in liver injury. We found that DMCs administration alleviated liver fibrosis and restored the liver function in fibrotic mice induced by CCl_4_. Furthermore, in an acute irradiation induced damage model, DMCs could engraft into the liver tissue for a long period and promoted the survival of mice exposed to 8Gy lethally total-body irradiation. These discoveries provide important evidence that DMCs therapy has a beneficial effect on liver injury, and provide new insight into liver injury therapy depending on the alternative cells.

## Materials and Methods

### Mice

**Figure 1:**
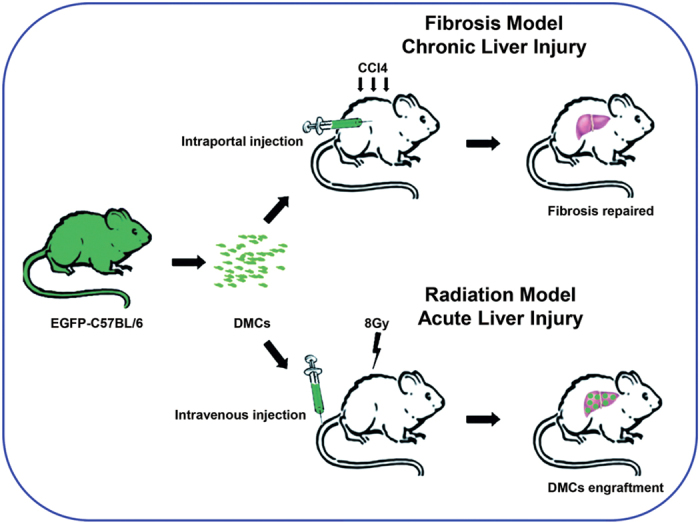
Experimental design. In the current study, two different animal models were used to investigate potential therapeutic effect of DMCs transplantation in liver injury. The results demonstrated that DMCs administration could alleviate liver fibrosis and restored the liver function in fibrotic mice induced by CCl_4_, and could engraft into the liver tissue for a long period and promoted the survival of mice exposed to 8 Gy lethally total-body irradiation.

EGFP-transgenic C57BL/6 donor mice (CByJ.B6-Tg(CAG-EGFP)1Osb/J) were obtained from Model Animal Research Center of Nanjing University (AAALAC accredited)^17^. C57BL/6J male mice were obtained from Experimental Animal Center, Third Military Medical University (TMMU). All the mice were maintained in the animal facility at TMMU. All experimental protocols were approved by the Institutional Animal Care and Use Committee of TMMU, and were conducted in accordance with the “Animal Care and Use Committee Guidelines” of the university. All mice were housed under identical conditions and given food and water ad libitum. The experimental design is summarized in Fig. 1.

### DMCs preparation

The procedures of DMCs preparation were performed as previously described^18^. In brief, full-thickness skin samples were obtained from the dorsum of neonatal EGFP-transgenic C57BL/6 mice, digested at 4 °C overnight with 0.25% trypsin (Hyclone, China). Next, the dermis layer was dissociated by flushing with D-Hank’s solution, and the suspension was filtered through a nylon mesh to remove cellular debris and centrifuged. Finally, 1 × 10^7^ DMCs could be isolated from the dermis of one neonatal EGFP-transgenic C57BL/6 mouse, and then the single-cell suspension were infused intravenously without cultivation and proliferation *in vitro*.

### Induction of Hepatic Fibrosis and DMCs Transplantation

To induce hepatic fibrosis, intraperitoneal injections of CCl_4_ (0.6 ml/kg body weight diluted 1:10 in olive oil) were administered to mice twice per week for 10 weeks. Transplantation of DMCs (2 × 10^6^) was performed via the hepatic portal vein at week 6 of CCl_4_ treatment. Control groups were given PBS only. The animals were killed at 1 day, 3 day, 1 week, 2 weeks and 4 weeks after transplantation, and their liver samples and blood were collected for analysis.

### Hydroxyproline assay

To investigate the content of hepatic hydroxyproline, Snap-frozen liver specimens (200 mg) were weighed, hydrolyzed in NaOH. We quantified the hydroxyproline content according to the manufacturer’s instructions of the hydroxyproline detection kits (Nanjing Jiancheng, China).

### Sirius red staining

To identify the extent of histological fibrosis, the liver fibrosis area was quantified with sirius red staining. In brief, sections were incubated in picrosirius red (Sigma, USA) for 2 h and washed with acetic acid and water.

### Histologic and immuneassy

Livers tissues were fixed in 10% neutral buffered formalin, embedded in paraffin, cross sectioned, and 4 mm in thickness were dewaxed and rehydrated. The transplanted dermal derived cells in the liver were identified by the GFP (1:100, Beyotime, China). To explore the phenotypes of the donor cells, the cellular surface markers were examined with immunofluorescence technique as described previously^19^. For the immunofluorescence examination, liver tissues were incubated with 5% bovine serum albumin and then incubated with primary antibody overnight at 4 °C. Primary antibodies were as follows: PCK (1:100; abcam, USA), F4/80 (1:100; abcam, USA), Ki67 (1:100; Thermo, USA), VEGFR2 (1:100; abcam, USA), vimentin (1:100; abcam, USA), CD45 (1:100; Biolegend, USA), α SMA (1:100; abcam, USA). Slides were stained with DAPI and then examined by fluorescence microscope. The bar of the picture at 200 magnification represent 100 μm and at 400 magnification represent 50 μm.

### The DMCs detection rate in organs of transplanted mice

At time point of 1, 3, 5, 7, 10, 14, 30, 60, 90, 120 and 270 days after DMCs engraftment respectively, the heart, liver, spleen, lung, kidney, bone marrow and intestine of each animal were harvested. Tissues were fixed, embedded in paraffin and sectioned. For each organ, 6 sections were randomly selected and the dermal derived cells were identified by GFP. With GFP positive cells detected, the DMCs were considered to be engrafted in the organ. And the detection rate of each organ was calculated as following: no. of mice with engrafted cells in the organ/total no. of detected mice.

### Morphometric analysis

Stained slides were random and a minimum of 10 serial, nonoverlapping fields were photographed at 200 magnification. For GFP, Ki67 and PCK assay, the mean positive cell number of one field was measured using using Image J software (NIH, Bethesda, USA), and the relative number was displayed with the ratio between the number of the tansplantation group and the control group. For collagen I and Sirius red assessment, the percentage staining of the total field was measured using Image J software.

### Serum Analysis

Biochemical parameters were measured using standard clinical methods. The peripheral blood was collected via cardiac puncture for the liver function assay after 4 weeks of transplantation. And the alanine aminotransferase (ALT), aspertate aminotransferase (AST) and albumin levels of the blood samples were detected using an Automated Chemical Analyzer in the Southwest Hospital (Chongqing, China) according to the manufacturer’s instructions.

### C-reaction protein (CRP) measurement

Serum of mice was respectively collected 3 days and 7 days after radiation, as well as the normal mice. The collected each sample was detected with an enzyme-linked immunosorbent assay (ELISA) kit for CRP concentrations. We did a pre-test to determine the optimal sample diluted concentration, then we added 100 ul diluted sample per well to measure CRP level according to the manufacturer’s instructions (Boster, China). Each sample was run in duplicate.

### Western blot analysis

Total proteins from liver tissue were analyzed by western blot analysis as previously described^20^. The proteins were extracted and resolved by SDS-PAGE. Then proteins were electroblotted onto PVDF membrane and blocked with fat-free milk. Blots were probed with 1:1,000-diluted primary antibodies were incubated overnight at 4 °C. Blots were then incubated with secondary antibody. Finally, the proteins were visualized by enhanced chemiluminescence exposure to X-ray film according to the manufacturer’s instructions. The blots were scanned, and the gray scale measurement was performed on the scanned images using Image J software.

### Survival study of irradiated mice treated with non-cultured DMCs

Recipient adult C57BL/6 mice were treated with a lethal dose of irradiation with a dosage of 8Gy at 0.985Gy/min using a cobalt-60 irradiator. DMCs cell suspension was prepared for transplantation by resuspension in PBS. PBS or DMCs (at 2 × 10^6^ cells, total volume of 100 μl) were slowly infused via the tail vein 4 hours after irradiation. The mice were randomized to receive PBS or DMCs. Mice were placed back in cages in a temperature-controlled room (22 °C) with 12-h light and dark cycles immediately after treatment. We monitored survival rate every day for 90 days.

### *In vivo* endocytosis assay

Two months after DMCs transplantation, fluorescent latex beads (Sigma, 20 μL beads in total volume of 200 μl) were injected into the irradiated mice through tail vein. Mice were sacrificed 2 hours after injection. The liver tissues were harvested and frozen sectioned. Slides were examined by laser scanning confocal microscopy (Carl Zesis).

### Statistical analysis

Data were expressed as mean ± SEM. Survival data were compared using the Kaplan-Meier test (Prism 5.0; Graphpad Software).An independent-samples t test was used to determine the significant differences between two groups. P < 0.05 was considered statistically significant.

## Results

### DMCs alleviated liver fibrosis and restored the liver function in fibrotic mice induced by CCl_4_

**Figure 2:**
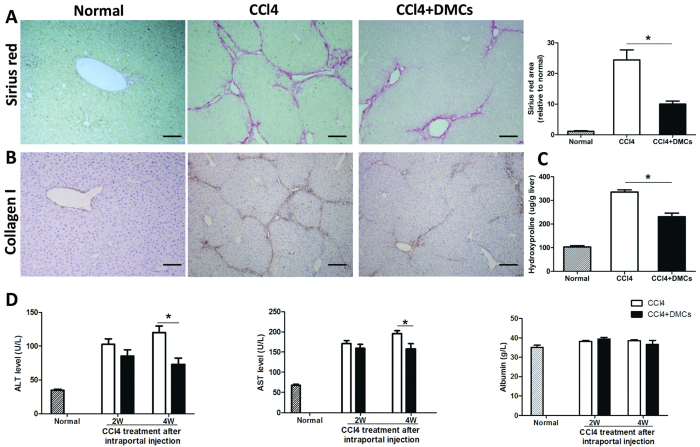
DMCs alleviated liver fibrosis and restored the liver function in fibrotic mice induced by CCl_4_. (**A**) DMCs therapy significantly alleviated fibrosis in chronic liver fibrosis mice detected by Sirius red staining 4 weeks later. (**B**) Hepatic collagen I expression was reduced in the transplanted mice compared to the controls. Bar represent 100 μm (200× magnification). (**C**) Hepatic collagen concentration, measured by the hydroxyproline assay, was significantly reduced in the transplanted mice compared to the controls. Bar represent 100 μm (200× magnification). (**D**) Liver enzymes ALT/AST and serum albumin were monitored 2 weeks and 4 weeks after DMCs transplantation in mice. Serum analysis suggested serum ALT and AST levels were significantly decreased in the transplanted mice compared with controls four weeks after DMCs treatment. n = 6–7 per group. Error bars represent means ± SEM. *Indicates Independent-Samples t-test p-values < 0.05.

We and others previously proved that DMCs were capable of homing to injuried tissues and stimulate wound repair response^11^,^13^,^14^,^18^,^21^,^22^,^23^,^24^,^25^, such as spinal cord injury, chronic nerves injury, Parkinson disease, skin wound, skeletal muscle injury, GVHD disease and sepsis. In this study we investigate for the first time whether DMCs could restore the liver structure and function in the experimental model of liver fibrosis. We established the chronic injury model of liver fibrosis by CCl_4_ administration which is thought to be the most closely resembling that of human liver cirrhosis. DMCs were transplanted into liver via the hepatic portal vein after 6 weeks of CCl_4_ administration. The results revealed that DMCs therapy significantly alleviated fibrosis in chronic liver fibrosis mice detected by Sirius red staining 4 weeks later (P < 0.05, Fig. 2A). Moreover, we also investigated whether hepatic collagen expression could be changed by DMCs therapy. As illustrated in Fig. 2C, hepatic collagen concentration, measured by the hydroxyproline assay, was significantly reduced in the transplanted mice compared to the controls. And this effect was also confirmed by the decreased collagen I staining (Fig. 2B). These results all indicated that the DMCs could alleviate the liver fibrosis. Furthermore, the liver enzymes ALT/AST and serum albumin were monitored after DMCs transplantation in mice (Fig. 2D). We found that administrated with DMCs reduced the serum ALT and AST levels two weeks after transplantation, although there were no significant differences. However, four weeks after DMCs delivery, the serum ALT and AST levels were significantly decreased in the transplanted mice compared with controls, which gave the evidence that DMCs transplantation showed a positive effect on the CCl_4_ injured liver *in vivo*.

### The therapeutic mechanism of DMCs on liver fibrosis by transient engraftment

**Figure 3:**
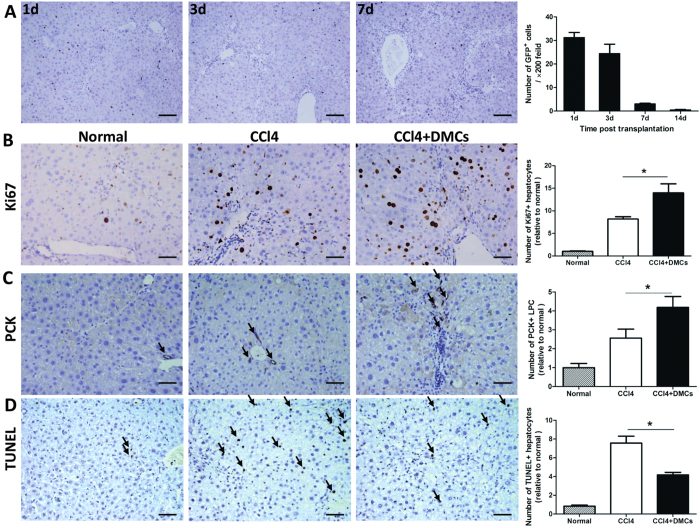
The detection and biological character of transient engraftment DMCs in liver. (**A**) After the delivery of DMCs, the mean number of engrafted cells was detected under light microscope by GFP detecting. The number of transplanted DMCs decreased gradually with time passing. n = 5 for each time point. Bar represent 100 μm (200× magnification). (**B**) The liver specimens were detected with Ki67, and the results showed that Ki67 positive hepatocytes cells significantly rose up after DMCs transplantation. Bar represent 50 μm (400× magnification). (**C**) PCK positive cells in DMCs recipients significantly increased on day 7. Bar represent 50 μm (400× magnification). (**D**) The liver sections were assayed with TUNEL staining, and quantification analysis showed that the number of TUNEL-positive cells significantly declined after DMCs transplantation. Bar represent 50 μm (400× magnification). n = 6 per group. Error bars represent means ± SEM. *Indicates Independent-Samples t-test p-values < 0.05.

**Figure 4:**
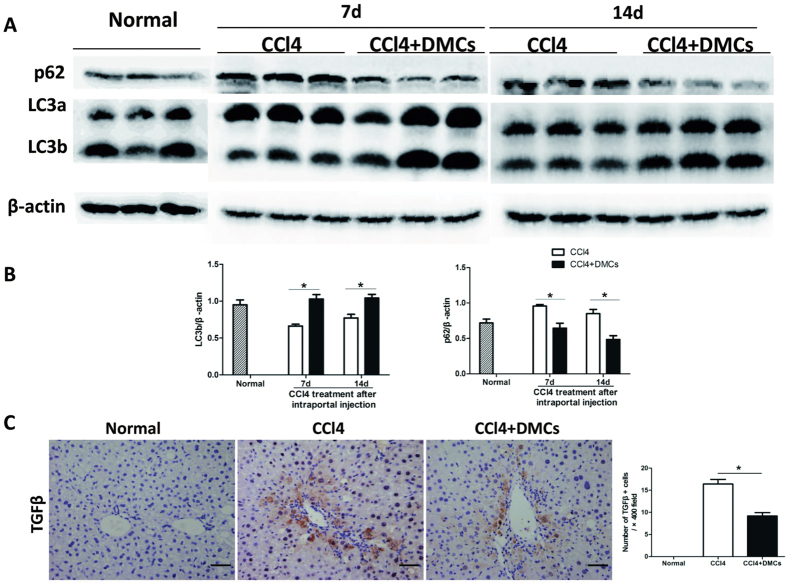
Autophagy induced by DMCs in the CCl_4_-damaged liver. (**A**) Western blots showing the protein levels of autophagic signaling markers LC3 and p62 in the liver tissues. (**B**) Densitometric quantification of LC3 and p62 in liver extracts. (**C**) Liver specimens were detected with TGFβ, and the results showed that TGFβ positive hepatocytes cells significantly declined after DMCs transplantation. Error bars represent means ± SEM. *Indicates independent-samples t-test p-values < 0.05. Bar represent 50 μm (400× magnification).

To investigate the role of DMCs on chronic liver injury, we observed the cell engraftment on the liver of the model induced by CCl_4_. After the delivery of DMCs, the mean number of engrafted cells was detected under light microscope by GFP detecting. Liver specimens were obtained at 1, 3, 7 and 14d after cell delivery. The statistics showed that the number of transplanted DMCs decreased gradually with time passing (Fig. 3A), and the transplanted DMCs could not be detected 4 weeks after delivery. Although DMCs were transiently engrafted in the liver after transplantation, the dramatic attenuation of liver fibrosis was observed. The effect of transiently engrafted donor cells could be amplified by paracrine signaling to host cell populations as reported by Thomas, J. A. *et al*.^26^ or indirect effects on liver as reported by Mouiseddine *et al*.^27^. To further investigate the function of DMCs on liver regeneration, the liver specimens were detected with Ki67, a marker of proliferation throughout the cell cycle. The results showed that Ki67 positive cells significantly rose up after DMCs transplantation (Fig. 3B). And cells expressing PCK, a sensitive marker for liver progenitor cells, significantly increased in DMCs recipients on day 7 (Fig. 3C). These results implied that DMCs therapy improved liver regeneration. The antiapoptotic effect of DMCs were also examined by the TUNEL staining of the liver specimens. Figure 3D shown that liver apoptosis was significantly decreased in mice from DMCs transplantation group compared with mice from the control group. Because autophagy is reported to be activated and play a role in the regenerative potential of stem cell therapy^28^,^29^. Autophagy was further examined in order to identify the mechanism of action of DMCs on liver regeneration. We examined the expression of autophagic signaling markers LC3b and p62 by Western blot. Figure 4A shown that LC3b was high expressed in DMCs transplanted mice compared to control mice and p62 degradation revealed a decrease in DMCs transplanted mice compared to control mice. Recently, autophagy has been suggested to be involved in fibrosis by modulating TGF-β expression^29^. Meanwhile, TGF-β is considered as one of the main molecular agents inducing fibrosis^30^. We stained liver tissue for the detection of TGF-β and found that the increased TGF-β expression was observed in both CCl_4_ control mice and DMCs treatment mice. However, the DMCs engraftment could significantly decrease the degree of elevation (Fig. 4C). Taken together, the net effect of transplanted DMCs was not only a reduction in fibrosis but also improved regeneration of the injured liver.

### DMCs preferentially engrafted into the liver tissue and improved the survival in lethally total-body irradiated mice

**Figure 5:**
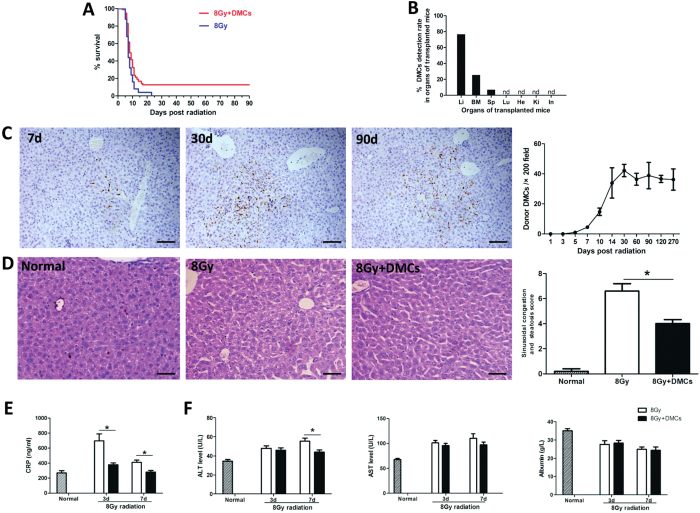
DMCs mainly engrafted into the liver tissue, improved the survival and decreased liver damage in lethally total-body irradiated mice. (**A**) DMCs treatment significantly reduced mortality in lethally total-body irradiated mice. Kaplan-Meier curves represent survival rate in the 2 groups. Survival rate was significantly higher in the DMCs group (12.621%, n = 94) than the PBS group (0 ,n = 25). (**B**) Transplanted DMCs preferentially engrafted into the liver tissue (DMCs detection rate: 76.7%). Transplanted DMCs were sporadically observed in hematopoietic tissues including the bone marrow of 11 mice (DMCs detection rate: 25.5%) and the spleen of 3 mice (DMCs detection rate: 6.9%). Li = liver, BM = bone marrow, Sp = spleen, Lu = lung, He = heart, Ki = kidney, In = intestine. (**C**) The engrafted GFP positive cells can be detected in liver, and the GFP positive cells quantities showed a significant increasing tendency with time. Bar represent 100 μm (200× magnification). n = 3 for each time point. (**D**) Histological examination of liver biopsy specimens was used to assess the injury of irradiated liver. At 2 weeks after radiation, the sinusoidal congestion and steatosis score of DMCs group were much lower compared with control group. Bar represent 50 μm (400× magnification). (**E**) The CRP levels were elevated after lethally total-body irradiation in mice, and the DMCs engraftment could significantly decrease the degree of elevation. (**F**) The AST/ALT levels decreased after the DMCs engraftment, and the serum ALT level were significantly low especially 7 days after irradiation. n = 5 per group. Error bars represent means ± SEM. *Indicates Independent-Samples t-test p-values < 0.05.

In addition to cirrhotic liver model by CCl_4,_ we also used an acute irradiation induced liver damage to investigate the therapeutic potential of DMCs since large dose of irradiation was demonstrated to injury the liver^31^,^32^. The data showed that transplantation of DMCs promoted the survival of mice exposed to 8 Gy lethally total-body irradiation, which implied potential protective effect of DMCs for irradiation injury. Through continuous observation for 90 days, we found that mice administrated with dermal cells after TBI exhibited 12.6% higher survival rate than that of the control group (p < 0.05) (Fig. 5A). To further study the engraftment of the transplanted DMCs, transplanted mice were acquired and different organ tissues were harvested. The result showed that the liver had the significantly highest detection rate of engrafted DMCs (76.7%) compared with other organs, and there were also transplanted DMCs sporadically observed in hematopoietic tissues including the bone marrow of 11 mice (25.5%) and the spleen of 3 mice (6.9%) (Fig. 5B). Additionally, we observed the number of GFP positive cells respectively in liver, bone marrow and spleen. The engrafted cells can be detected in all liver samples after 5 days post-transplantation. The engraftment efficiency was extremely low in bone marrow and spleen. However, the number of detected cells in liver showed a significant increasing tendency with time (Fig. 5C). Additionally, histological examination of liver biopsy specimens was used to assess the injury of irradiated liver. Liver irradiation resulted in typical sinusoidal congestion and hepatic steatosis. At 2 weeks after radiation, the sinusoidal congestion and steatosis score for control group was 6.6 ± 0.6 (Fig. 5D). However, these changes were much less pronounced in mice transplanted with DMCs (4.0 ± 0.3, P < 0.5), suggesting transplantation of DMCs could improve the repair of radiation-induced liver injury. The serum levels of CRP were elevated after lethally total-body irradiation in mice, and the DMCs engraftment could significantly decrease the degree of elevation (Fig. 5E), which exhibited a certain anti-inflammatory effect. Meanwhile, we detected the liver function in irradiated mice to measure liver damage. The results indicated that the AST/ALT levels decreased after the DMCs engraftment, and the serum ALT level were significantly low especially 7 days after irradiation (Fig. 5F).

### Engrafted DMCs exhibited both macrophage-like feature and mesenchymal cells property in the irradiated mice

#### Engrafted DMCs exhibited macrophage-like phenotype

**Figure 6:**
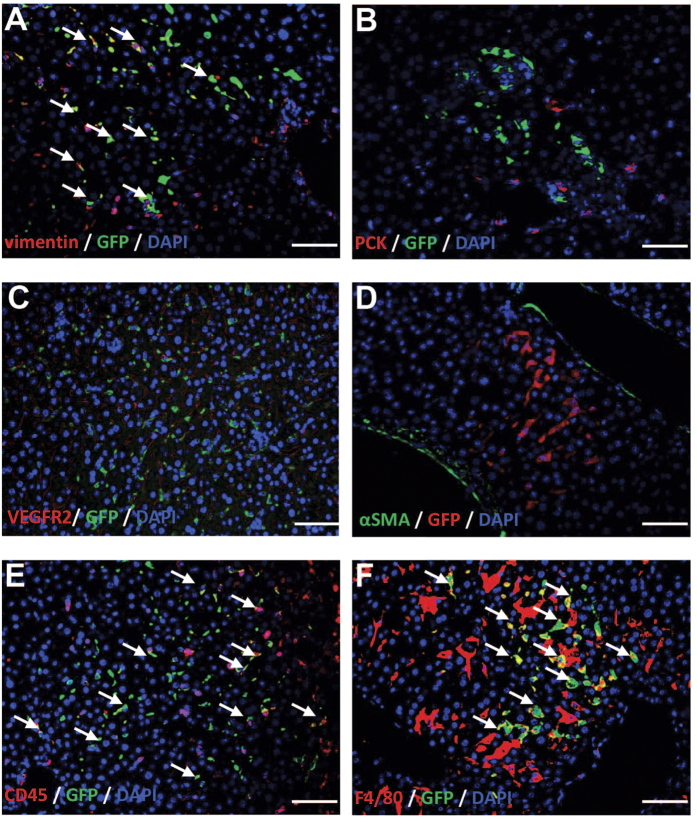
Identification of engraftment DMCs in liver. To further explore what phenotypes the GFP positive cells exhibited, donor dermal cells in the liver of transplanted mice were investigated by immunofluorescence. All GFP positive cells engrafted in the liver were labeled with vimentin, which implied that they were originated from dermis (**A**). As followed, to detect whether these cells displayed markers of liver cells, we incubated them with markers of liver progenitor cells (PCK), sinusoid endothelial cells (VEGFR2), and hepatic stellate cells (αSMA) respectively and the results showed none of these molecules expressed (**B–D**). However, all GFP positive cells presented the hematopoietic marker CD45 and macrophage surface maker F4/80 (**E,F**). Colocalization (arrows) of the GFP (green) with vimintin (**A**), CD45 (**E**) and F4/80 (**F**) staining respectively indicates that DMCs were expressing vimintin, CD45 and F4/80.Bar represent 50 μm (400× magnification).

**Figure 7:**
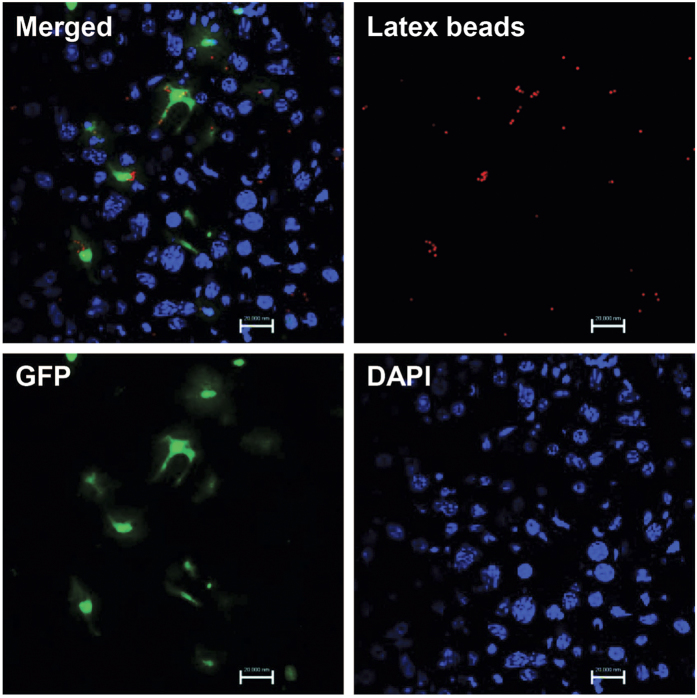
Phagocytosis assay of engrafted cells. Four hours after latex beads administration, phagocytosis ability was analyzed by fluorescence microscope. Engrafted cells in the transplanted irradiated mice could phagocytize latex beads. Bar represent 20 μm (630× magnification).

To further explore what phenotypes the GFP positive cells exhibited, donor dermal cells in the liver of transplanted mice were investigated by immunofluorescence (Fig. 6). To avoid an epidermal contamination when dermal cells suspensions were prepared, we detected dermal cells with mesenchymal maker (vimentin). The result showed that all GFP positive cells engrafted in the liver were labeled with vimentin, which implied that they were originated from dermis. As followed, to detect whether these cells displayed markers of liver cells, we incubated them with markers of liver progenitor cells (PCK), sinusoid endothelial cells (VEGFR2), and hepatic stellate cells (αSMA) respectively and the results showed none of these molecules expressed. However, all GFP positive cells presented the hematopoietic marker CD45 expression. More importantly, the double immunofluorescence suggested that all GFP positive cells expressed macrophage surface maker F4/80. According to the character that these GFP positive cells located in the sinusoid and most of them showed elongated cell morphology, we suggested that these implanted cells should be the macrophage-like cells. To further study whether the settled dermal macrophage-like cells showed the functional potential, we carried out the phagocytosis assays by administrating the transplanted irradiated mice with latex beads intravenously. Four hours after administration, dermal macrophage-like cells in the transplanted irradiated mice was found to phagocytize latex beads, indicating that settled dermal cells exhibited the basic function of macrophages (Fig. 7).

#### Engrafted DMCs demonstrated proliferative property *in vivo* and *in vitro*

**Figure 8:**
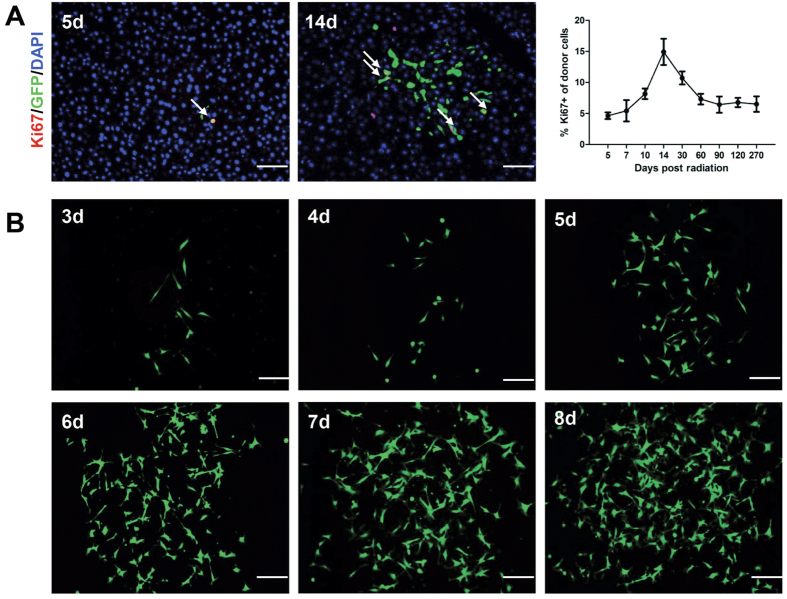
Engrafted DMCs demonstrated proliferative property *in vivo* and *in vitro*. (**A**) The liver specimens were detected with Ki67 at several time points to detect proliferative property of the engrafted cells. At day 5, the donor dermal cells were found to locate within or closely appose the hepatic vessels. With the increasing number of engrafted cells detected, the percentage of Ki67 positive cells grew. n = 3 for each time point. Error bars represent means ± SEM. Bar represent 50 μm (400× magnification). (**B**) The engrafted cells were isolated and cultured *in vitro*. The GFP positive cells displayed elongated cell morphology, showing active proliferation ability. Bar represent 100 μm (200× magnification).

To further uncover the biological character of the settled dermal macrophage-like cells, the liver specimens were detected with Ki67 at several time points. At day 5, the donor dermal cells were discovered to locate within or closely appose the hepatic vessels. With the increasing number of engrafted cells detected, the percentage of Ki67 positive cells grew (Fig. 8A). The result demonstrated that the settled donor dermal cells retained potential proliferation property. Meanwhile, we isolated and cultured the cells from the liver tissue of the transplanted mice. As illustrated in Fig. 8B, the GFP positive cells displayed elongated cell morphology. The primary passage of the cell population showed active proliferation ability, and presented with no growth retardation after subculture. These results suggested that donor dermal cells well preserved the mesenchymal cells-owned properties of self–renewal both *in vivo* and *in vitro*.

#### Neither BM macrophages nor RAW264.7 improved survival in lethally irradiated mice

**Figure 9:**
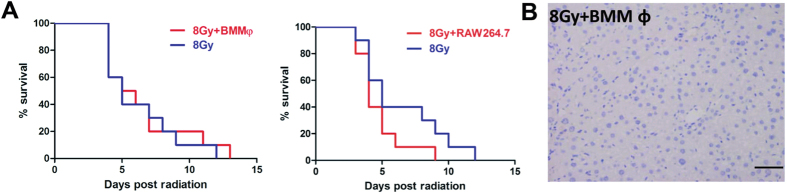
Neither BM macrophages nor RAW264.7 improved survival in lethally irradiated mice. (**A**) Neither BM macrophages nor RAW264.7 treatment reduced mortality in lethally total-body irradiated mice. Kaplan-Meier curves represent survival rate in the 2 groups. (**B**) The engrafted GFP positive cells cannot be detected in liver. Bar represent 50 μm (400× magnification).

In view of the long-term engraftment of macrophage-like cells derived from dermis in the recipient liver and a survival advantage of this transplantation after lethally radiation, we investigated whether BM macrophages (BMMφ) and RAW264.7 macrophages could show beneficial effects after radiation injury. However, the results suggested that neither BMMφ nor RAW264.7 therapy exhibited survival promotion in lethally irradiated mice (Fig. 9A). Meanwhile, transplanted BMMφ (GFP+) could not be detected in the irradiated liver, which was different from the engraftment of dermal derived macrophage-like cells (Fig. 9B). These results suggest that a unique population of dermal cells exhibiting properties of both macrophages and mesenchymal cells is involved in the repair of liver injury after total body irradiation.

## Disscussion

Liver is an important organ with highly regenerative capacity and involved in various activities, including bile acid excretion, production of blood clotting factors, destruction of bacteria in the blood and detoxification. Therefore, acute and chronic liver injury could be caused by many factors such as xenobiotics, drugs and microbes, when they were metabolized by liver. Unfortunately, patients with end-stage liver diseases showed relatively poor prognosis. Due to negative feasibility of liver transplantation, research and application of alternative cell therapy has been developed emergently and regarded as new hopes for replacement therapy for liver diseases.

Currently, adult stem cells are main sources for cell therapy. Researchers have shown that bone marrow–derived mesenchymal stem cells effectively rescued experimental liver failure and contributed to liver regeneration^5^,^32^, and this offers a potentially alternative therapy to organ transplantation for treatment of liver diseases^32^. More than 30 clinical trials are registered worldwide for evaluating MSC therapy for fibrosis (http://clinicaltrials.gov). Liver and pulmonary fibrosis are most widely represented. In most of these studies, only organ functionality is evaluated. Thus, it is not clear whether the improvement of the symptoms and quality of life is due to fibrosis reduction or the amelioration of other pathological features^30^. Compared to MSCs, different site-specific mesenchymal cells also show regenerative and immunomodulatory properties for a cell therapy candidate. Due to the abundant location and easy acquirement, stem cells from epidermis and hair follicle have been well studied. However, stem cells of the skin dermis didn’t not been widely focused. Until recently, several studies reported that dermis of human and murine harbored a couple of stem cell subpopulations with multi-lineage differentiation capacity^33^,^34^. Recently, it has been demonstrated that dermal fibroblast was used to generate functional hepatocytes that could repopulate mouse livers by reprogramming to endoderm progenitor cells without dedifferentiation into iPSCs^35^. Furthermore, our recent work indicated that the incidence and severity in a mouse model of acute GVHD decreased after transplantation of non-cultured dermal derived cells^13^. All these studies indicated that dermal dervied cells could be a key source of stem cells for regenerative medicine.

However, it remains to be clarified if dermal derived mesenchymal cells are able to directly contribute to the rescue of liver injury. Furthermore, their ability to respond to different type of liver injury has not been investigated. Thus, in the current study, we used two different animal models to investigate potential therapeutic effect of DMCs transplantation in liver injury. Firstly, we used CCl_4_ induced-liver fibrosis mice model to investigate the function of DMCs in treating chronic liver damage. Hepatic fibrosis is a progressive pathological process involving multicellular and molecular events that ultimately lead to the deposition of excess ECM proteins such as collagens. Results from preclinical and clinical trials highlight the ability of MSCs to act on fibrosis through different mechanisms: (i) immunosuppression, (ii) inhibition of the TGF-β1 pathway, (iii) reduction of hypoxia and oxidative stress, and (iv) restoration of ECM degradation. Thus, the potential of MSC therapy lies in the ability to act simultaneously on various fibrogenesis parameters^30^. This study evaluated fibrosis using Sirius Red staining and IHF for collagen I. We found that DMCs transplantation improved CCl_4_ induced-liver fibrosis companied with decrease of AST and ALT. Although engrafted DMCs in the chronic liver injury decreased in the fibrosis liver, DMCs could be detected for four weeks after transplantation. This phenomenon implied that DMCs showed a transient engraftment in chronic fibrosis liver. In our present study, we also found that DMCs therapy improved liver regeneration after CCl_4_ induced damage. It has been suggested that autophagy may play a role in the regenerative potential of stem cell therapy. Park, M found that Tonsil-derived mesenchymal stem cells ameliorate CCl_4_-induced liver fibrosis in mice via autophagy activation^29^. Autophagy activation appears to be involved in DMCs -mediated tissue regeneration as well, as shown by an increase in LC3b expression and decrease in p62 expression. However, the exact underlying mechanism needs further elucidation.

Furthermore, we used an acute irradiation induced liver damage to investigate the therapeutic potential of DMCs. The data showed that transplantation of DMCs promoted the survival of mice exposed to 8 Gy lethally total-body irradiation, which implied potential protective effect of DMCs for irradiation injury. Unexpectedly, the transplanted cells were mainly engrafted in the liver. Donor DMCs were primarily found to locate within or closely to hepatic vessels on 5 days after transplantation, and these cells, with potent proliferation capacity, increased almost 10 times at day 30. These donor DMCs could be detected even at 9 month after transplantation and still showed widespread distribution just as that at day 14. Although previous studies reported that endothelial progenitor cells, mesenchymal stem cells and human amniotic epithelial cells could locate into the liver transiently after cell transplantation, our work firstly proved that dermal derived cells could efficiently implant in liver after intravenous transplantation. Furthermore, several previous studies reported hepatic-like cells could be induced from dermal cells^15^,^16^,^36^,^37^,^38^,^39^. However, we investigated the effects of dermal cells therapy for acute liver injury *in vivo* and found no hepatocytes differentiated from dermal cells in our present study. Interestingly, we identified a unique dermal cell subpopulation with a phenotype of macrophages with the property of phagocytosis and local proliferation in the liver tissues of survival mice. And we also found both BM macrophages and RAW264.7 cells could not locate into liver and improve the survival time of irradiated animals. Furthermore, our findings indicated that the mechanism of action of DMCs in treating acute liver damage is partly through upregulated autophagy. These finding may open an new avenue for cell therapy of liver injury.

## Conclusion

In summary, we used two different animal models to investigate potential therapeutic effect of DMCs transplantation in liver injury. We found that DMCs administration could alleviate liver injury both in chronic liver fibrosis model induced by CCl_4_ and acute irradiation induced damage model. Furthermore, a unique population of DMCs could engraft into the liver tissue for a long period, exhibiting both mesenchymal and macrophage-like cell phenotype, and improve the survival of mice exposed to 8 Gy lethally total-body irradiation. Our findings demonstrate that DMCs therapy has a beneficial effect on liver injury for the first time, providing new insight into liver injury therapy depending on the alternative cells.

## Additional Information

**How to cite this article**: Tan, L. *et al*. Contribution of dermal-derived mesenchymal cells during liver repair in two different experimental models. *Sci. Rep*. **6**, 25314; doi: 10.1038/srep25314 (2016).
